# Chronic Coccygodynia and ganglion impar block: How does contrast material distribution affect treatment outcomes?

**DOI:** 10.1111/papr.70024

**Published:** 2025-03-29

**Authors:** Yucel Olgun, Savas Sencan, Sena Unver, Nuride Osmanli, Serdar Kokar, Osman Hakan Gunduz

**Affiliations:** ^1^ Division of Pain Medicine, Department of Physical Medicine and Rehabilitation Agri Training and Research Hospital Agri Turkey; ^2^ Division of Pain Medicine, Department of Physical Medicine and Rehabilitation, Faculty of Medicine Marmara University Istanbul Turkey; ^3^ Division of Pain Medicine, Physical Medicine and Rehabilitation Department, Faculty of Medicine Sakarya University Sakarya Turkey; ^4^ Department of Physical Medicine and Rehabilitation, Faculty of Medicine Marmara University Istanbul Turkey

**Keywords:** coccygodynia, contrast material distribution pattern, ganglion impar, ganglion impar block, treatment success

## Abstract

**Aim:**

To assess the influence of contrast material distribution patterns on treatment success in patients with chronic coccygodynia undergoing ganglion impar block (GIB).

**Methods:**

An evaluation was conducted on 58 patients who underwent GIB from August 2021 to August 2023 at a university hospital's interventional pain management center. Numeric rating scale (NRS) scores were recorded before the procedure and at 1‐month post‐procedure. The patients were categorized into two groups based on treatment success, defined as at least a 50% reduction in the NRS score at 1 month.

**Results:**

There were no significant differences between the two groups regarding age, gender, BMI, symptom duration, comorbidities, coccyx curvature type, presence of anterior/posterior subluxation, presence of posterior spicule, type of approach, contrast distribution direction, and contrast dye level. Patients with coccygodynia experienced statistically significant benefits from GIB treatment at the 1‐month follow‐up (*p* < 0.001).

**Conclusion:**

Although the use of contrast material in fluoroscopic procedures is the gold standard to prevent possible complications, the distribution pattern of contrast does not significantly affect the success of GIB treatment in patients with coccygodynia. Further prospective and long‐term follow‐up studies are required to validate these findings.

## INTRODUCTION

Coccygodynia, characterized by pain in the coccygeal region, predominantly affects women and is associated with obesity.^1^ The most important etiological factors in the formation of coccygodynia are external and internal traumas. While conservative treatments can alleviate symptoms in most patients, some experience persistent pain, leading to a significant decline in quality of life. In such cases, interventional treatments become crucial.^1^, ^2^

The ganglion impar, positioned at the sacrococcygeal junction or within the coccyx, is the solitary ganglion within the bilateral paravertebral sympathetic chains, situated in the retroperitoneal region. It provides nociception and sympathetic innervation to the perineum and terminal urogenital organs.^1^, ^3^ In the cadaveric dissection study by Oh et al.,^3^ which aimed to show the topographic anatomy of the ganglion impar, its localization was found at the sacrococcygeal joint level in 18% of cases. Other localizations, according to the relative index, were determined by drawing a line from the midpoint of the sacrococcygeal joint to the tip of the coccyx. The ganglion impar localizations were as follows based on the relative index: 0.1 (8%), 0.2 (18%), 0.3 (26%), 0.4 (20%), 0.5 (8%), and 0.6 (2%). Additionally, it was shown that as the size of the coccyx increased, the localization of the ganglion impar shifted more caudally.

Ganglion impar block (GIB) is an effective and safe treatment frequently used for chronic coccygodynia, a cause of pelvic pain.^2^ GIB can be performed through different approaches, including paramedian, midline, anococcygeal ligament, sacrococcygeal or intercoccygeal joint spaces, or lateral approaches.^4^ Under fluoroscopic guidance, the needle is advanced through the sacrococcygeal disc to the anterior joint, with contrast material injection resulting in the characteristic reversed comma sign, although this pattern may not be present in every case.^5^, ^6^

Various demographic, clinical, and radiological (e.g., coccyx curvature types, fracture history, subluxation) factors that may affect GIB treatment success have been investigated.^7^, ^8^, ^9^ On the contrary, to our knowledge, the effect of the contrast spread pattern on the treatment success of patients with coccygodynia has not been investigated. Contrast agents in fluoroscopy help confirm procedural accuracy and detect possible complications. According to our clinical experience and literature, the distribution pattern of contrast material is decisive in terms of the treatment success of fluoroscopy‐guided interventional pain procedures. Lee et al.^10^ showed that the saddle‐type contrast distribution pattern positively affected the treatment outcome in patients who received transforaminal epidural steroid injections due to sciatica. In another study by Reuschel et al.,^11^ the effect of contrast material distribution on treatment in CT‐guided periradicular injections was evaluated, and the contrast material distribution pattern and periradicular injections were evaluated. They found that the circumferential, large contrast distribution pattern positively influenced the treatment outcome in patients who underwent CT‐guided periradicular injections.

We have not identified any studies that demonstrate whether the contrast distribution pattern affects the treatment outcome, either positively or negatively, in patients undergoing GIB. The objective of this study is to investigate the impact of contrast medium distribution patterns during the procedure on the treatment success of GIB in patients with chronic coccygodynia.

## METHODS

### Study design and participants

This retrospective study adhered to the Declaration of Helsinki's principles and obtained written informed consent from all participants. This study conforms to all STROBE guidelines and reports the required information accordingly. After receiving institutional ethics committee approval, we retrospectively evaluated patients who underwent fluoroscopy‐guided GIB between August 2021 and August 2023 at a tertiary hospital's pain management center.

**FIGURE 1:**
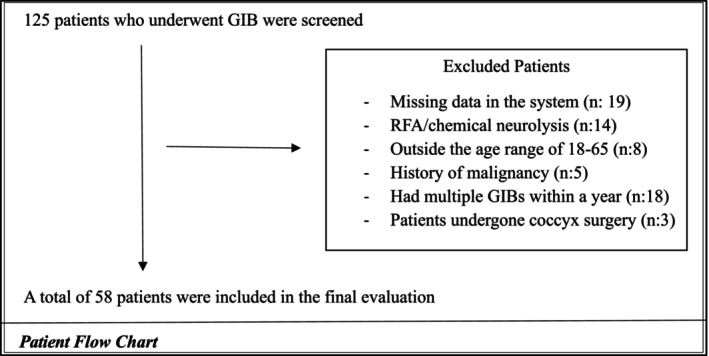
Patient flow chart.

**FIGURE 2:**
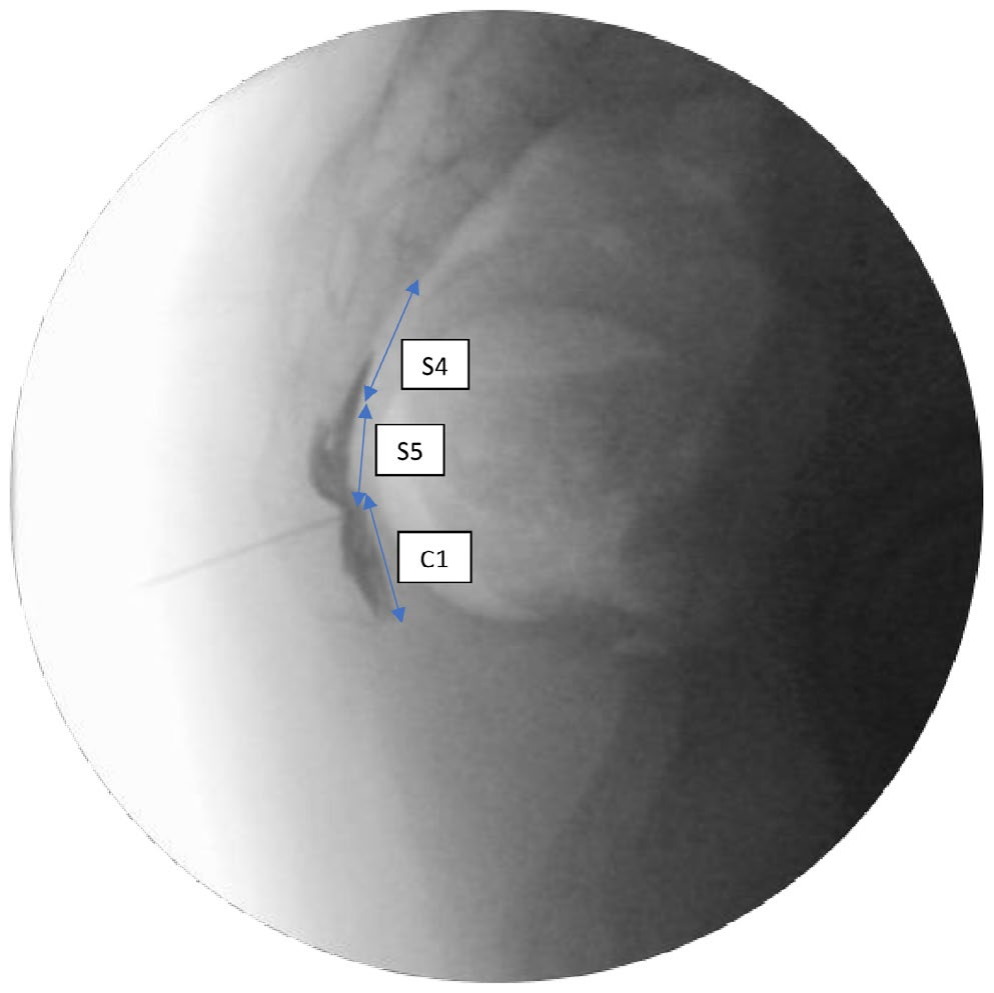
Contrast distribution spreading over three vertebral levels (lateral view).

**FIGURE 3:**
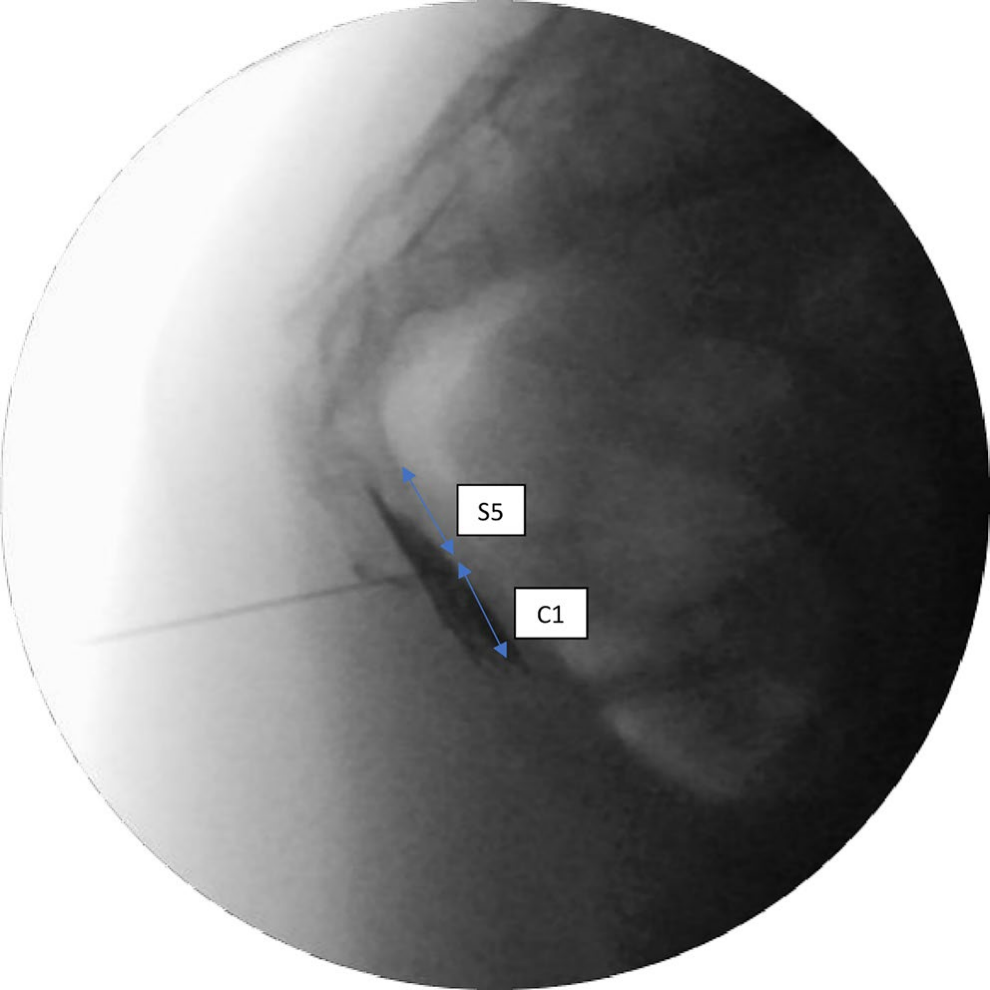
Contrast distribution spreading over two vertebral levels (lateral view).

**FIGURE 4:**
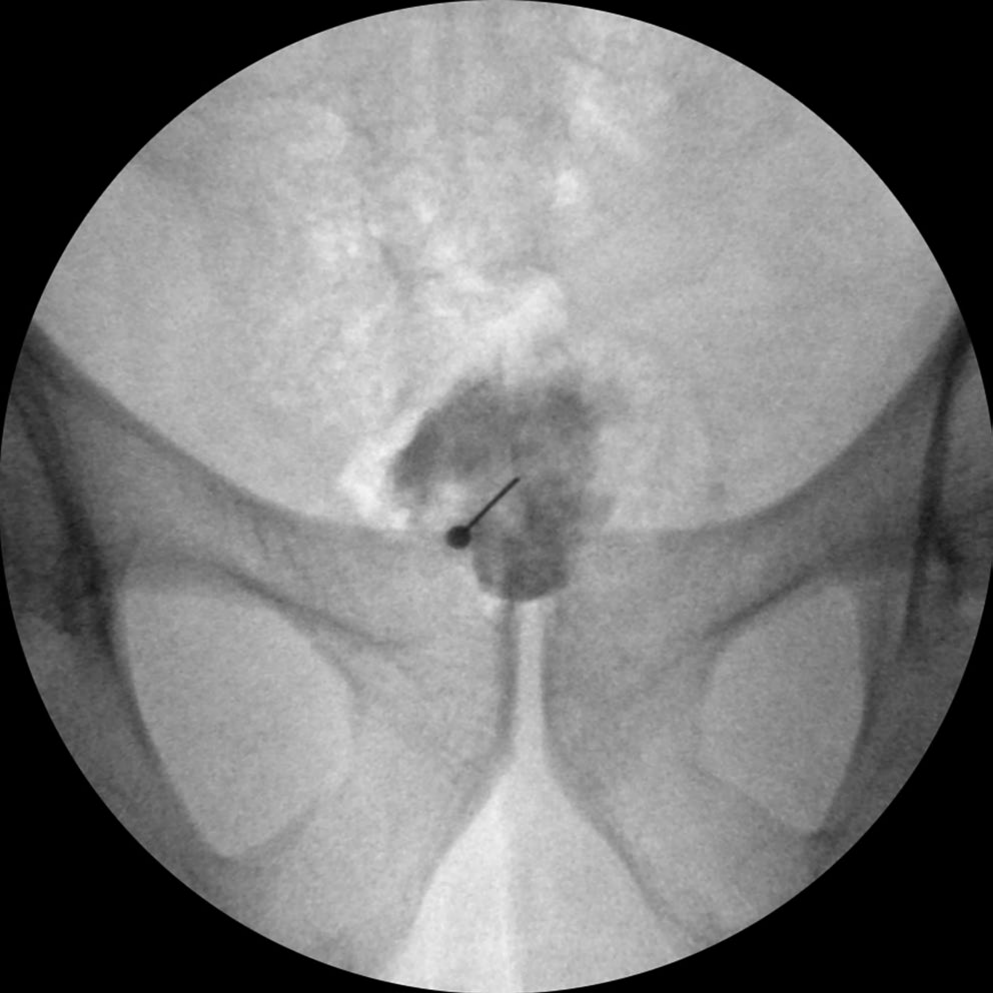
Contrast distribution in the anteroposterior (AP) view.

A total of 125 patients who underwent GIB were scanned from the hospital system. After applying the exclusion and inclusion criteria, this study was conducted with 58 patients who underwent ganglion impar injections for chronic coccygodynia. The inclusion criteria included patients who underwent GIB between the ages of 18–65 in the study. Patients without relevant data or who received injections other than GIB, patients with contrast material allergy, patients with malignancy, RFA (radiofrequency ablation)/chemical neurolysis, coccyx fracture, those who had undergone coccyx surgery, and patients who underwent multiple GIBs within a year were excluded from the study (Figure 1). The contrast distribution direction was divided into cephalad, caudad, and both directions. The level of contrast distribution was determined according to the number of coccyx and sacral vertebrae over which it spreads (Figures 2, 3, 4).

### Data collection

We collected patient data from hospital records, including demographic information, Numeric Rating Scale (NRS) scores, symptom duration, trauma history, presence of anterior/posterior subluxation, presence of posterior spicule, coccyx curvature type, type of approach (transsacrococcygeal/transcoccygeal), contrast distribution direction, level of contrast distribution, CCI (Charlson Comorbidity Index), and treatment success were also noted. The patients' NRS scores were recorded before the procedure, at 1 h post‐procedure, and at 1 month follow‐up. Patients were categorized into groups based on treatment success, defined as at least a 50% reduction in NRS score at 1 month.

### Intervention procedures

The patients were positioned in the prone position with a pillow placed under their lower abdomen. The area was disinfected three times with povidone‐iodine and then covered with a sterile drape that had an opening positioned over the injection site. The sacrococcygeal and intercoccygeal joints were visualized in lateral imaging via fluoroscopy. A local anesthetic (3 mL of 2% prilocaine) was administered into the skin and subcutaneous tissue. Subsequently, the tip of a 22‐gauge Quincke spinal needle was carefully advanced towards the ganglion impar with intermittent fluoroscopic guidance. The needle was further advanced through the vertebral disc until its tip was positioned just anterior to the ventral sacrococcygeal ligament, indicated by a loss of resistance. The correct placement of the needle tip was verified by injecting 1 cc of iohexol contrast dye into the retroperitoneal space for all patients. After confirming that the contrast spread to the ganglion impar without any vascular distribution, a 5‐cc mixture of the injectate (6 mg betamethasone acetate, 3 cc 0.5% bupivacaine, and 1 cc saline) was administered. Patients were then monitored in the observation room for potential complications for 1 h before being discharged with instructions.

All procedures were carried out by a pain medicine specialist with over 10 years of experience. The Ziehm Vision R fluoroscopy unit was used for intermittent imaging throughout the procedure, following the acquisition of both written and verbal consent from the patients. Linear and circular collimation were used in all procedures to minimize radiation exposure in accordance with the ALARA principle.

### Statistical analysis

Statistical analysis was conducted using SPSS version 27.0.1 (IBM Corp., Armonk, NY). Continuous data were summarized as mean (standard deviation) or median (interquartile range), while categorical data were reported as counts and percentages. Chi‐square or Fisher's exact tests were applied for categorical data. The normality of quantitative data was assessed using the Shapiro–Wilk test. For comparisons, the Mann–Whitney *U* test was employed for non‐normally distributed data, and the independent *t*‐test was used for normally distributed data. Statistical significance was set at a *p*‐value < 0.05.

## RESULTS

A total of 58 patients, 42 (72.4%) of whom were women, were included in the study. The mean age was 43.1 years, and the symptom duration averaged 34.25 months (range: 1–120). Of the patients, 19% were using duloxetine and 12% were using gabapentinoids. Pre‐procedural mean NRS score was 8.05 ± 1.24, reducing to 1.05 ± 2 1 h after the procedure and 4.01 ± 2.88 1 month post‐procedure. The transsacrococcygeal approach was used in 44 cases (75.9%), whereas the transcoccygeal approach was used in 12 cases (24.1%). The distribution levels of contrast media as one‐level, two‐level, and three‐level were 12 (20.7%), 31 (53.4%), and 15 (25.9%), respectively. The most common distribution levels of contrast media were found to be two levels with 31 procedures (%53.49). The distribution direction of contrast media as cephalad, caudad, and both directions were 12 (20.7%), 3 (5.2%), and 43 (74.1%), respectively.

When comparing patients based on treatment success, no significant differences were found between age, gender, BMI, CCI, coccyx type, presence of anterior/posterior subluxation, symptom duration, trauma history, presence of anterior/posterior subluxation, presence of posterior spicule, coccyx curvature type, type of approach, contrast distribution direction, and contrast dye level (Table 1). In one patient, vascular uptake of contrast material was noted. After repositioning the needle, the contrast distribution was typical. No additional side effects or complications were reported.

**TABLE 1:** Comparison of groups in terms of treatment success.

	Treatment success group (*n* = 41)	Treatment failure group (*n* = 17)	*p* value
Age (years)	42.17 ± 13.65	45.35 ± 10.81	0.396
BMI (kg/m^2^)	27.36 ± 5.94	28.25 ± 6.22	0.611
Symptom duration (months)	34.21 ± 26.64	34.35 ± 34.28	0.987
CCI	0.87 ± 1.51	0.75 ± 1.52	0.776
PreNRS	8.07 ± 1.36	8.0 ± 0.93	0.841
PostNRS (1 h)	0.75 ± 1.46	1.4 ± 2.59	0.227
PostNRS (1 month)	2.04 ± 1.32	6.76 ± 1.64	<0.001
Gender			
Male	12 (29.3%)	4 (23.5%)	0.755
Female	29 (70.7%)	13 (76.5%)	
Trauma history			
Yes	18 (44%)	5 (29.4%)	0.384
No	23 (56%)	12 (70.6%)	
Type of approach			
Transsacrococcygeal	32 (78%)	12 (70.6%)	0.737
Transcoccygeal	9 (22%)	5 (29.4%)	
Coccyx type			
Type 1	5 (12.2%)	5 (29.4%)	0.370
Type 2	8 (19.5%)	4 (23.5%)	
Type 3	15 (36.6%)	6 (29.4%)	
Type 4	12 (29.3%)	2 (11.8%)	
Type 5	1 (2.4%)	0 (0%)	
Type 6	0 (0%)	0 (0%)	
Anterior subluxation			
Yes	2 (4.9%)	0 (0%)	0.892
No	39 (95.1%)	17 (100%)	
Posterior subluxation			
Yes	9 (22%)	2 (88.2%)	0.480
No	32 (78%)	15 (11.8%)	
Posterior spicule			
Yes	2 (4.9%)	1 (5.9%)	0.875
No	39 (95.1%)	16 (94.1%)	
Contrast distribution direction			
Cephalad	10 (24.4%)	2 (11.8%)	0.323
Caudad	3 (7.3%)	0 (0%)	
Both directions	28 (68.3%)	15 (88.2%)	
Contrast dye level			
One‐level	10 (24.4%)	2 (11.8%)	0.512
Two‐level	20 (48.8%)	11 (64.7%)	
Three‐level	11 (26.8%)	4 (23.5%)	
Contrast dye level (single/multiple)			
Single level	10 (24.4%)	2 (11.8%)	0.478
Multiple level	31 (75.6%)	15 (88.2%)	

Abbreviations: BMI, body mass index; CCI, Charlson comorbidity index; NRS, numeric rating scale.

## DISCUSSION

GIB has been demonstrated as an effective and safe treatment option for patients with coccygodynia.^2^, ^8^, ^12^, ^13^ The present study investigated the effect of contrast distribution patterns on the success of GIB treatment. It was found that contrast dye level and distribution direction were similar between treatment success and treatment failure groups. No major complications occurred in the study, but vascular involvement was noted in one patient, and it was resolved by adjusting the needle position.

In the current study, the 1‐month pain scores of 58 patients with coccygodynia were evaluated retrospectively. Pain scores at the 1‐h and 1‐month post‐procedure were significantly lower than preprocedural pain scores. According to the treatment success criteria, 60.3% of our patients achieved treatment success at the 1‐month follow‐up. This result aligns with previous studies on GIB in patients with chronic coccygodynia.^2^, ^13^ Datir et al. reported no major complications in their GIB study conducted with CT, while Malhotra et al. observed similar findings with fluoroscopy, with all patients demonstrating high tolerance for the procedure.^13^, ^14^ In our study, no major complications were detected, and only one patient (1.7%) experienced a minor complication, in which vascular uptake of the contrast medium was noted and resolved after repositioning the needle. These findings underscore the effectiveness of GIB as a therapeutic modality with a low incidence of complications.

Coccydynia is frequently encountered as a musculoskeletal disorder with resistant pain, sometimes accompanied by neuropathy. In a study by Sencan et al.^15^ comparing the GIB to caudal epidural block, 52.9% of the patients had concomitant neuropathic pain. Although both treatments were effective, GIB was more effective in the short term. The innervation of the coccyx is rich and complex, making the exact mechanism of GIB's effect in treating coccydynia a subject of debate.^4^, ^7^ The sympathetic nervous system plays a significant role in pain pathways at multiple levels. Some experimental studies suggest the sympathetic nervous system can regulate peripheral inflammation and nociceptive activation.^16^ In coccydynia, chronic irritation of the coccygeal nerves leads to increased sensitivity in the ganglion impar and the somatosensory system.^17^ Besides sympathetic blockage, GIB may provide a secondary effect by blocking the anterior branches of the coccygeal nerves that pass through the sacrococcygeal joint.^18^ Additionally, studies have shown that blocking around the sacrococcygeal joint, the posterior sacrococcygeal ligament, and the coccygeal nerve is effective in controlling pain in coccydynia.^19^, ^20^

Steroids and local anesthetics are used to reduce the inflammatory response and pain caused by chronic irritation. These drugs suppress inflammation by their anti‐inflammatory effects and washout, removing inflammatory substances. Both medications reduce the conduction of chronic pain signals and thus play a role in preventing pain sensitization. Additionally, steroids show strong anti‐inflammatory effects through membrane stabilization, blocking ectopic signals, and inhibiting neuropeptide synthesis. Local anesthetics provide temporary antinociception by blocking sodium channels.^21^, ^22^, ^23^ Sencan et al.^12^ demonstrated that using local anesthesia alone in GIB produced favorable results, but when combined with steroids, the effects lasted longer.

The fact that the majority of the patients in our study were female and in the middle‐aged group aligns with findings from other studies.^24^, ^25^, ^26^ In a retrospective study of 106 patients, Kim et al.^9^ reported that demographic data such as age, gender, and BMI did not affect treatment success. Similarly, Sencan et al.^8^ found no relationship between treatment success and age, gender, and BMI. Consistent with these findings, our results indicate that age, gender, and BMI did not impact treatment success.

Plancarte^27^ first described GIB, in which the needle tip is advanced through the anococcygeal ligament and up to the anterior surface of the sacrococcygeal ligament. However, it is crucial to note that this approach carries a significant risk of inadvertent rectal perforation.^28^ The paramedian double‐bent needle approach, once commonly used for GIB, has fallen out of favor due to technical challenges.^29^ Although various alternative approaches for GIB have been documented, fluoroscopy‐guided transsacrococcygeal and transcoccygeal techniques are now the most widely used methods.^13^

GIB was also performed using ultrasound and CT guidance.^30^, ^31^ However, ultrasound cannot replace fluoroscopy due to the necessity of fluoroscopy, which is essential for determining safe depths and accurate injection sites.^30^ The ganglion impar is not visible on fluoroscopy and is challenging to visualize even with MRI. As a result, contrast material is crucial for confirming its location. It ensures precise needle placement and helps identify complications, such as rectal perforation, that may arise during the procedure. Consequently, using contrast material under fluoroscopic guidance is considered the gold standard for GIB.^32^ The present study examined the impact of contrast material distribution patterns, a key element of fluoroscopy‐guided GIB, on treatment success. The results demonstrated that neither the direction of contrast distribution (cephalad/caudad) nor the contrast dye level influenced treatment outcomes. Therefore, we think that contrast distribution patterns alone are inadequate for evaluating treatment success.

The transsacrococcygeal approach is often preferred for GIB due to its ease of performance.^25^, ^33^ However, when ossification or fusion of the sacrococcygeal disc obstructs access, the first intercoccygeal joint may be used as an alternative.^25^, ^34^, ^35^ Both approaches carry risks, including discitis, bleeding, and rectal perforation.^36^ In our study, no major complications were observed, with only one patient (1.7%) experiencing a minor complication.

The ganglion impar exhibits anatomical variations in its location. Oh et al.^3^ demonstrated in cadaver dissections that it is typically positioned closer to the first intercoccygeal joint than to the sacrococcygeal joint. In our study, we selected the joint space that appeared most clearly on lateral fluoroscopic imaging. The procedure was performed at the sacrococcygeal joint in 75.9% of patients, while the remaining patients underwent it at the first intercoccygeal joint. Malhotra et al.^13^ also reported that both the transcoccygeal and transsacrococcygeal approaches were safe and effective for GIB. Our findings similarly suggest that both approaches yield comparable and effective treatment outcomes.

### Limitations

This study has several limitations. First, it is a retrospective study with short‐term results. Another limitation is that the number of patients is relatively small but similar to the number of patients in ganglion impar block studies in the literature. The strength of the study is that, to the best of our knowledge, it is the first study to investigate the effect of contrast distribution patterns on GIB treatment success.

## CONCLUSIONS

Contrast distribution patterns (contrast distribution level/direction) do not significantly affect the success of GIB in treating coccygodynia. Further prospective and long‐term follow‐up studies are required to validate these findings.

## AUTHOR CONTRIBUTIONS

Yucel Olgun: Writing/manuscript preparation; Sena Akca, Nuride Osmanli: Investigation; Serdar Kokar: Computation; Savas Sencan: Study conception, review, and editing; Osman Hakan Gunduz: Supervision.

## CONFLICT OF INTEREST STATEMENT

The authors declare that they have no conflict of interest.

## PATIENT CONSENT STATEMENT

Written informed consent was obtained from all participants.

## Data Availability

The datasets supporting the findings of this study are available from the corresponding author upon reasonable request.
